# MultiVI: deep generative model for the integration of multimodal data

**DOI:** 10.1038/s41592-023-01909-9

**Published:** 2023-06-29

**Authors:** Tal Ashuach, Mariano I. Gabitto, Rohan V. Koodli, Giuseppe-Antonio Saldi, Michael I. Jordan, Nir Yosef

**Affiliations:** 1 Center for Computational Biology, University of California, Berkeley, CA USA; 2 Department of Electrical Engineering and Computer Sciences, University of California, Berkeley, CA USA; 3 Department of Statistics, University of California, Berkeley, Berkeley, CA USA; 4 Allen Institute for Brain Science, Seattle, WA USA; 5 Department of Systems Immunology, Weizmann Institute of Science, Rehovot, Israel

**Keywords:** Software, Machine learning, Transcriptomics, Data integration

## Abstract

Jointly profiling the transcriptome, chromatin accessibility and other molecular properties of single cells offers a powerful way to study cellular diversity. Here we present MultiVI, a probabilistic model to analyze such multiomic data and leverage it to enhance single-modality datasets. MultiVI creates a joint representation that allows an analysis of all modalities included in the multiomic input data, even for cells for which one or more modalities are missing. It is available at scvi-tools.org.

## Abstract (web summary)

By learning a joint representation using deep generative modeling, MultiVI integrates multimodal and single-modality single-cell datasets, which enhances multiple functionalities.

## Main

The advent of technologies for profiling the transcriptional and chromatin accessibility landscapes at a single-cell resolution has been paramount for cataloging cellular types and states^1,2^. However, most uses of single-cell RNA-sequencing (scRNA-seq)^3,4^ and single-cell assay for transposase-accessible chromatin with sequencing (scATAC-seq)^2,5^ have been limited such that a given cell can only be profiled by one technology. Recently, multimodal single-cell protocols have emerged for simultaneously profiling gene expression, chromatin accessibility and, more recently, the abundance of surface protein in the same cell^6,7^. This concomitant measurement enables a more refined categorization of cell states and, ultimately, a better understanding of the mechanisms that underlie their diversity.

The emerging area of multimodal profiling has benefited greatly from new statistical methods that jointly account for multiple data types in a range of analysis tasks^8–10^. Another promising application of multimodal assays, however, is to improve the way in which the more common and less costly single-modality datasets (for example, scRNA-seq) are analyzed and interpreted. By leveraging datasets with multimodal (paired) information, one can infer properties of the missing modalities and thus gain new insight that is otherwise difficult to achieve. To provide a comprehensive solution, such an integrative analysis should be carried out at two levels. First, it should generate a low-dimensional summary of the state of each cell that reflects all the input molecular types, regardless of which type of information is available for that particular cell. As commonly done in other applications of single-cell genomics, such a representation can facilitate the identification of subpopulations or gradients and enable a more informative data visualization^11^. A second level of analysis should generate a normalized, batch-corrected view of each high-dimensional data type (for example, accessibility of each chromatin region), either observed or inferred. Such an analysis can enable broader identification of molecular features that characterize cellular subpopulations of interest.

Here, we introduce MultiVI, a deep generative model for probabilistic analysis of multimodal datasets, which also enables their integration with single-modality datasets. Focusing on gene expression and chromatin accessibility as our main case study, we demonstrate that MultiVI provides solutions for the two levels of analysis, with a low-dimensional summary of cell state and a normalized high-dimensional view of both modalities (measured or inferred) in each cell. MultiVI was designed to account for the general caveats of single-cell genomics data, namely batch effects, different technologies for the same modality, variability in sequencing depth, limited sensitivity and noise. It does so while explicitly modeling the statistical properties of each modality, treating the discreteness of the scRNA-seq signal and the binary nature of the scATAC-seq signal. A key part in the design of MultiVI is its modularity, which allows for inclusion for additional data modalities. Here, we demonstrate it by adding surface protein expression with tagged antibodies as a third modality^6,7^. The extended model accounts for properties of the protein data (for example, nonzero background component), and enables integration and joint analysis with single-modality (RNA-, chromatin- or protein-only) datasets.

A recent method (Cobolt^12^) presented an approach similar to that of MultiVI, with promising results. As we will show, MultiVI provides a more comprehensive solution for integrating and interpreting information across modalities, studies and technologies (a summary of all the computational experiments performed in the paper is available in Supplementary Fig. 1). In addition to showcasing its ability to derive accurate low-dimensional representations, we demonstrate several key properties of MultiVI as a way of imputing high-dimensional signals. First, we demonstrate that MultiVI provides calibrated estimates of the uncertainty in the imputed values (for example, predicted chromatin accessibility for scRNA-seq only cells and predicted gene expression for scATAC-seq only cells), such that less accurate predictions are also less confident. Second, we demonstrate that these estimates of uncertainty give rise to accurate estimates of differential gene expression or chromatin accessibility in cells for which the respective modality was not available. Third, we show that even if a population of cells has information from only one modality, accurate imputation may still be achieved when multimodal information is available for related populations (thus effectively performing out-of-sample prediction). MultiVI is available in scvi-tools as a continuously supported, open source software package, along with detailed documentation and a usage tutorial at https://docs.scvi-tools.org/.

## Results

### The MultiVI model

MultiVI leverages our previously presented variational autoencoding (VAE^13^) models for gene expression (scVI, ref. ^14^), chromatin accessibility (PeakVI, ref. ^15^) and protein abundance (totalVI, ref. ^16^). For clarity, we focus the discussion here on jointly modeling scRNA- and scATAC-seq data. The extension to surface protein measurements is provided in the Methods section.

Given multimodal data from a single cell (*X*), and a sample (or batch) *S*, we divide the observations into gene expression $\left({X}_{\mathrm{R}}\right)$ and chromatin accessibility $\left({X}_{\mathrm{A}}\right)$. Two deep neural networks, termed encoders, learn modality-specific, batch-corrected multivariate normal distributions that represent the latent state of the cell based on the observed data, *q*(*z*_R_∣*X*_R_, *S*) and *q*(*z*_A_∣*X*_A_, *S*), from the expression and accessibility observations, respectively. To obtain a latent space that reflects both modalities, we penalize the model so that the distance between the two latent representations is minimized and then estimate the integrative cell state *q*(*z*∣*X*_R_, *X*_A_, *S*) as the average of both representations. The states of cells for which only one modality is available (that is, unpaired), are drawn directly from the representation for which data are available (that is, *z*_R_ or *z*_A_). This encoding part of the model can be naturally extended for handling other molecular properties (such as protein abundance), by including additional encoder networks.

In the second part of the model, observations are generated from the latent representation using modality-specific decoder neural networks. Similar to our previous models for gene expression (scVI) and accessibility (PeakVI), RNA expression data are drawn from a negative binomial distribution and the accessibility data from a Bernoulli distribution. The likelihood is computed from both modalities for paired (multimodal) cells, and only from the respective modality of unpaired cells. Finally, during training, we include an adversarial component that penalizes the model if cells from different modalities are overly separated in latent space (Methods).

**Fig. 1:**
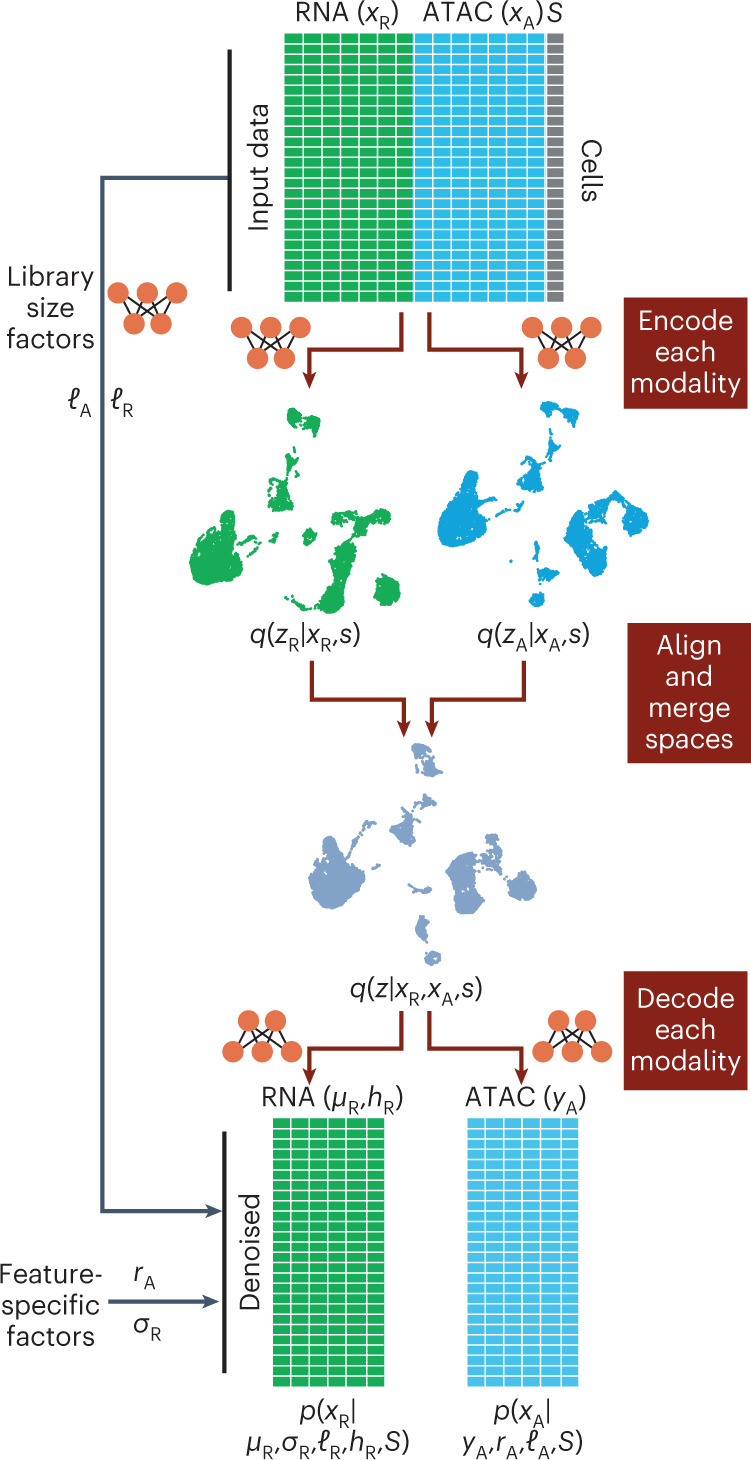
Conceptual model illustration in which input data (top) consist of chromatin accessibility (ATAC), gene expression (RNA) or both data types (multiome). Variable *S* represents experimental covariates, such as batch or experimental condition. Each data modality is encoded into modality-independent latent representations (using neural network encoders) and then, these representations are merged into a joint latent space. The joint latent representation is used to estimate (decode) the input data together with chromatin region-specific effects (*r*_A_), gene-specific dispersion (*σ*_R_), cell-specific effects (*ℓ*_A_, *ℓ*_R_), accessibility probability estimates (*Y*_Z_) and mean gene expression values (*μ*_R_).

This two-part architecture leverages the paired data to learn a low-dimensional representation of cell state, which reflects both data types, and it allows cells for which only one modality is available to be represented in the same (joint) latent space. Additionally, the generative part of the model provides a way to derive normalized, batch-corrected gene expression and accessibility values for both the multimodal cells (that is, normalizing the observed data) and for unpaired cells (that is, imputing unobserved data; Fig. 1 and Methods).

### MultiVI integrates paired and unpaired samples

We first trained MultiVI on a fully-paired peripheral blood mono-nuclear cells (PBMC) dataset from 10X genomics, and observed that our predicted library size factors (Methods) are highly correlated with the observed number of unique molecular identifiers in both the expression and accessibility libraries (Pearson’s correlation 0.97 and 0.91, respectively; Supplementary Fig. 1b,c). Next, to study how well MultiVI integrates paired and single-modality data into a common low-dimensional representation, we artificially unpaired the dataset. In this procedure, a random set of cells (between 1 and 99% of all cells) were unpaired such that each cell in the set appears twice: once with only gene expression data, and once with only chromatin accessibility data. This resulted in a heterogeneous dataset containing three sets of ‘cells’: one set has both modalities available, a second set with only RNA sequencing (RNA-seq) information and the third set with only ATAC-seq information.

Using these data, we compared MultiVI to Cobolt^12^, a model similar to MultiVI that uses products of experts to create a common latent space. To explore the performance of additional analysis strategies, we also added several adaptations of Seurat^8^. Specifically, we used the Seurat V4 code base with three different approaches: (1) gene activity, we converted the ATAC-seq data of the accessibility-only cells to gene activity scores (using the signac procedure), and then integrated all the cells using the gene-level data (that is, gene scores when RNA-seq is not available or gene expression when RNA-seq is available); (2) imputed, we followed the steps in (1) and then used Seurat to impute the RNA expression values for the accessibility-only cells and (3) weighted nearest neighbors (WNN), using WNN graphs, which leverage information from both modalities to create a joint representational space, then project single-modality data onto this space (Methods).

**Fig. 2:**
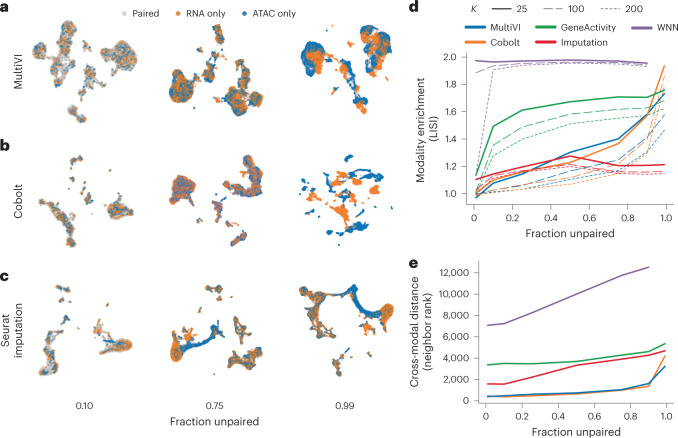
Integration of multiome paired and unpaired data. **a**–**c**, UMAP representations of the latent spaces learned by MultiVI (**a**), Cobolt (**b**) and Seurat using the RNA-imputation based integration (**c**), for various rates of unpaired data, colored by cell modality. **d**, Modality enrichment (LISI score), computed as the fraction of neighbors of the KNN that are from the same modality, normalized by the overall fraction of the cells from that modality. **e**, The mean distance between the two representations of artificially unpaired cells, measured as the number of cells between them.

We ran all methods on the artificially unpaired datasets and compared their latent representations (with the exception of the WNN-based approach on the 99% unpaired dataset, which failed to produce results due to the low number of paired cells; Fig. 2a–c and Supplementary Fig. 2). We first quantified the mixing performance by calculating the local inverse Simpson’s index (LISI) score described by ref. ^17^ (Fig. 2d). We found that algorithms based on generative modeling (Cobolt, MultiVI) outperform the alternative approaches of gene scoring and WNN in most rates of unpaired cells. Conversely, the Seurat-based imputation approach (unlike the other two Seurat-based approaches) maintains high mixing performance across all levels of unpaired cells. This result is expected, since each accessibility-only cell is represented by a gene expression vector that is an average over cells for which RNA-seq is available and that have gene expression profiles that are similar to one another (that is, a local neighborhood in a transcriptome-based space). It does not, however, indicate whether these representations are accurate.

We next examined the accuracy of the inferred latent space, taking advantage of the ground-truth information contained in our artificially unpaired datasets. Ideally, the two modality-specific representations of unpaired cells would be situated closely in the latent representation, as both capture the same biological state. We therefore looked at the distances between the two representations of each unpaired cell in the latent space created by each method. To account for the varying scales of different latent spaces, we used the rank distance (the minimal *K* for which the two representations are within each other’s *K*-nearest neighbors (KNN), averaged across all cells; Methods and Fig. 2e). In this experiment, we found that MultiVI and Cobolt maintain the multimodal mixing accuracy substantially better than the three alternatives, and that all methods have a deteriorating performance as the level of unpaired cells increases.

One of the key modeling decisions in MultiVI, compared with previous approaches such TotalVI (ref. ^16^), is the creation of latent representations for each data modality, followed by the calculation of an average representation, while penalizing the symmetric KL distance between representations. To test the robustness of this approach, we trained models with other approaches and compared them against default MultiVI. We tested replacing the symmetric KL divergence term with a maximum mean discrepancy term (MMD)^18^, as well as replacing the simple average with weighted averages in two settings: a global weighting scheme (*w*_m_, such that ∑_Modalities_*w*_m_ = 1) and a cell-specific weight (*w*_m,c_, such that ∑_Modalities_*w*_m,c_ = 1). In both cases, weights are learnable parameters optimized along with the rest of the model. We used two annotated multimodal datasets in which PBMCs are profiled using either DOGMA-seq^7^ or transcriptome-epitope-accessibility sequencing (TEA-seq)^6^ to evaluate latent space creation (Supplementary Fig. 3a,b). To evaluate each condition, we used the LISI metric together with several single-cell genomics batch correction and conservation of biological variation metrics, as defined in the scib package^19^ (Supplementary Information). In both datasets and under the different modeling decisions, all settings we tested yielded highly similar results (Supplementary Fig. 3c). We concluded that the model is robust with regard to these modeling decisions.

Taken together, these results show that the deep generative modeling approach, as embodied by MultiVI, effectively integrates unpaired scRNA and scATAC data while still preserving the biological state of each cell.

### Integration of independent studies

Our benchmark analyses in Fig. 2 rely on artificially unpaired data, where our model benefits from all data fundamentally being generated in a single batch and by a single technology. This does not reflect real-world situations in which it is desired to integrate datasets that were generated in different batches or even different studies. We therefore sought to demonstrate MultiVI on a set of real-world data. We collected three distinct datasets of PBMCs: (1) multimodal data from the 10X dataset we used previously; (2) ATAC-seq from a subset of hematopoiesis data generated by Satpathy et al.^20^, containing multiple batches of PBMCs as well as cell-type specific (FACS-sorted) samples and (3) PBMC data generated by several different technologies for scRNA-seq, taken from a benchmarking study by Ding et al.^21^. The datasets were processed to create a set of shared features (genes or genomic regions, when measurements are available), and annotations were collected from both the Satpathy et al. and Ding et al. datasets and combined into a shared set of cell-type labels (Methods). The resulting dataset has 47,148 (53%) ATAC-only cells from Satpathy et al., 30,495 (34%) RNA-only cells from Ding et al. and 12,012 (13%) jointly profiled cells from 10X.

**Fig. 3:**
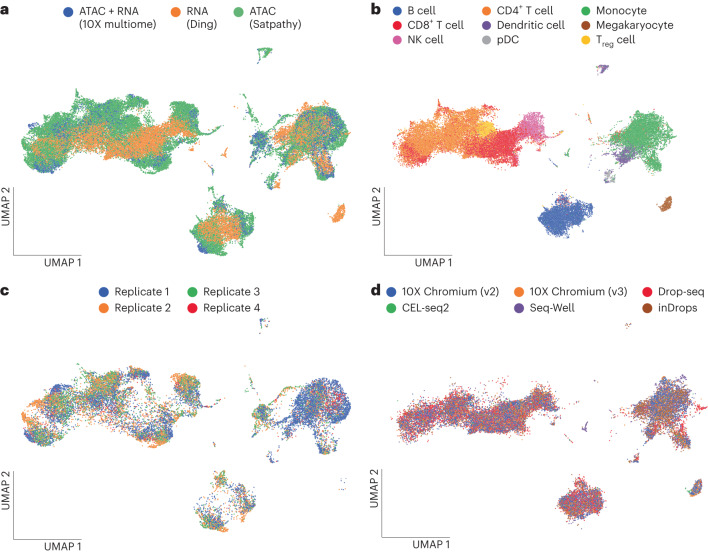
Integration of multiome data across technologies and replicates. **a**–**d**, UMAP representation computed from the latent space of MultiVI in which cells are color labeled by their modality (**a**) and cell-type label (**b**); scATAC-seq PBMC cells labeled by the replicate from which they were collected (**c**) and scRNA-seq cells labeled by their experimental technology (**d**). NK cell, natural killer cell; pDC, plasmacytoid dendritic cell; T_reg_ cell, regulatory T cell.

To gauge the extent of batch effects in these data, we ran MultiVI without accounting for the study of origin of each sample or its specific technology (which varies between the RNA-seq samples from Ding et al.). In this setting, cells stratified based on sample in the accessibility data, and based on technologies in the expression data, indicating that batch effects were affecting the latent representation (Supplementary Fig. 4). We then configured MultiVI to correct for batch effects and technology-specific effects within each dataset and re-ran the analysis (Methods). The resulting joint latent space mixes the three datasets well (Fig. 3a), while accurately matching labeled populations from both datasets (Fig. 3b). MultiVI achieves this while also correcting batch effects within the Satpathy data and technology-specific effects within the Ding data (Fig. 3c,d and Supplementary Fig. 5a–c). To study the correctness of the integration, we examined the set of labeled cells from the two single-modality datasets (FACS-based labels from Satpathy and manually annotated cells from Ding). For each cell, we identified its 50 nearest neighbors that came from the other modality and summarized the distribution of labels from the neighboring cells. We find a clear agreement between the labels of each cell and the labels of its cross-modality neighbors, with some mixing among related cell types (Supplementary Fig. 5d). This therefore demonstrates that MultiVI is capable of deriving biologically meaningful low-dimensional representations that effectively integrate data not only from different modalities, but also from different laboratories and technologies.

### Probabilistic data imputation with estimated uncertainty

**Fig. 4:**
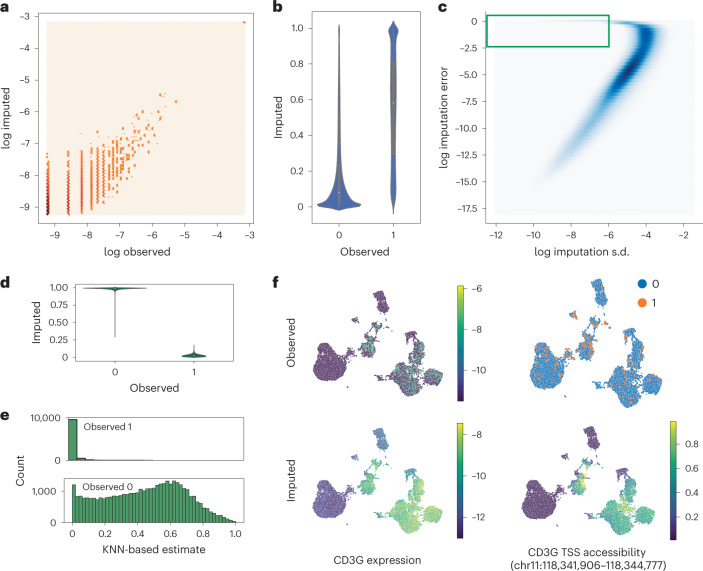
Imputation with uncertainty estimates. **a**, Normalized observed RNA counts by MultiVI-imputed RNA estimates; all values, including color intensity, are presented on a log scale (log(*x* *+* 1 − 4)) for stability. **b**, MultiVI-imputed accessibility estimates by the observed values. **c**, The imputation error (imputed − observed)^2^ as a function of the standard deviation (s.d.) of the imputed accessibility estimates. The green box marks the high-confidence–high-error values examined in the following panels. **d**, MultiVI-imputed accessibility estimates by the observed values for high-confidence–high-error cases. **e**, Smooth accessibility estimates for values observed as 1 (top) and 0 (bottom). Estimates computed by averaging the accessibility profiles of the 50 nearest neighbors, in a 50-dimensional space computed using latent semantic indexing. **f**, Observed and imputed values for CD3G expression and CD3G TSS accessibility. Expression values are normalized per cell and displayed on a log scale.

The generative nature of MultiVI enables several functionalities for analyzing the data in the full high-dimensional space, performing imputation of missing observations and modalities, estimation of uncertainty and differential analysis. To evaluate MultiVI’s data imputation capabilities, we resorted to the PBMC dataset from 10X (Methods) where 75% of the cells were artificially unpaired (as in Fig. 2). We used MultiVI to infer the values of the missing modality for the unpaired cells and found that for both modalities, the imputation had high correspondence to the observed values (Fig. 4a–c). Considering all the gene expression entries together, MultiVI achieves a Spearman correlation of 0.57 between the imputed values and the originally observed ones (scaled by library size). Since raw chromatin accessibility data are binary, we computed the area under the precision-recall curve to assess imputation accuracy, in which MultiVI reaches 0.41. Since the raw data can be markedly affected by low sensitivity, we also calculated the correlation between the imputed values and a smoothed version of the data (obtained with a method different from MultiVI; Methods), where the signal is averaged over similar cells (separately for ATAC-seq and RNA-seq), thus mitigating this issue. We obtained high level of correspondence between the imputed values and this corrected version of the raw data (Spearman correlations 0.8 and 0.86 for accessibility and expression, respectively; Supplementary Fig. 7a,b).

Next, we focused on uncertainty estimation for the imputed accessibility values. We measured the uncertainty of the model for each imputed accessibility value by sampling from MultiVI’s generative model (Methods) and found a strong relationship between the estimated uncertainty and the error at each data point, indicating that the model is indeed less certain of predictions that are farther from the hidden ground-truth values (Fig. 4c). Equivalent analysis for expression imputations is less informative due to the strong correlation between the average observed expression of a gene and the uncertainty of the imputed results.

We identified a small subset of chromatin accessibility values (roughly 0.5% of observations) for which we have high-confidence imputations that also have high error (Fig. 4c, green square). These high-confidence–high-error imputations correspond to cases where the model confidently predicts the opposite of the actually observed value (Fig. 4d). To investigate the source of these errors, we instead compared the imputed values to the smoothed version of the observed accessibility estimates (Methods). The smoothed data agree with the MultiVI predictions—namely, observations that were predicted as accessible tend to be open in highly similar cells, and observations that were predicted as inaccessible tend to be closed in similar cells (Fig. 4e). This indicates that these high-confidence high-error values may correspond to false negatives and false positives in the raw data.

As a specific example of imputation, we highlight the T cell marker gene CD3G. While the observed expression and the observed accessibility of the region containing the transcription start site (TSS) of the gene show high noise and sparsity, the imputed values are highly consistent and clearly mark the T cell compartment of the latent space (Fig. 4f).

To further test the usability of MultiVI’s imputation, we next explored a scenario in which the multi- and single-modal data come from different biological conditions. In this case, we resorted to the PBMCs dataset collected under the DOGMA-seq protocol^7^. In this dataset, PBMCs are profiled at a resting condition as well as after stimulation. We artificially created a dataset in which stimulated cells lack chromatin accessibility information and trained MultiVI in this dataset and the original (complete) one (Supplementary Fig. 6a,b). We first considered the resulting latent representations to evaluate the extent of batch-effect correction and preservation of biological information. To this end, we applied the scib^19^ metrics package on both the perturbed and complete dataset. We found that the scores of the perturbed dataset are similar (within 5%) to the scores of the unperturbed dataset (Supplementary Fig. 6c). Next, we assessed the accuracy of imputation of chromatin accessibility values in the stimulated cells by comparing the imputed values to the hidden accessibility values after smoothing the latter values, as above. In this test, we found accurate level of reconstruction (Spearman correlation 0.93; Supplementary Fig. 6d), highlighting MultiVI’s ability to infer missing modalities even under new perturbations.

Overall, these results show that MultiVI is capable of imputing missing observations effectively. The ability to quantify the uncertainty further allows the user to determine which imputed values are reliable for downstream analyses and which are not.

### Cross-modal differential analyses

Our results thus far demonstrate that MultiVI can be used to impute missing observations in situations where the multimodal and the single-modality data both contain similar cell types. The task becomes more challenging when considering a population of cells that is distinct from all other populations in the data and for which one of the modalities was not observed.

**Fig. 5:**
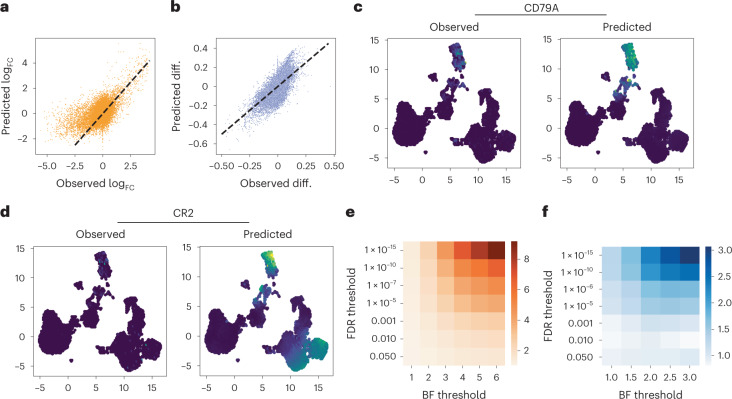
Differential analyses with missing modality. **a**,**b**, Differential effect sizes between B cells and the remainder of the data, comparing the effects computed from the held-out expression data with those predicted by MultiVI, for differential expression (**a**) and differential accessibility (**b**). **c**,**d**, Expression values for B cell marker CD79A (**c**) and B and T cell marker CR2 (**d**), observed in the held-out data (left) and predicted by MultiVI (right), displayed using latent space coordinated computed using all the available data. **e**,**f**, Fold enrichment of the overlap between statistically significant results for various significance thresholds for expression (**e**) and accessibility (**f**). Observed diff., observed differential. BF threshold, Bayes factor threshold.

To explore this, we used the same 10X PBMC dataset, with 75% of cells artificially unpaired, and clustered the latent space to identify distinct cellular populations (Supplementary Fig. 8a). We chose the B cell cluster, which we annotated as such using established markers (for example, CD19, CD79A). Next, we removed all expression information (paired or unpaired) from the B cell population, thus creating a distinct population for which only accessibility data are available to the model. In a second experiment, we removed all accessibility data from the B cell population to create a dataset for which only expression was observed in those cells (Supplementary Fig. 8b,c). We trained MultiVI separately on each of the two corrupted datasets, and used the model to perform differential analyses, comparing the B cell population and the remainder of the cells. Specifically, we conducted differential expression analysis with the model trained without B cell expression and differential accessibility with the model trained without B cell accessibility. Statistical significance was estimated with Bayes factor, as in previous work^14,15,22^ (Methods). To evaluate the accuracy of this analysis, we used standard differential analyses (not using generative models) on the held-out data to create ground-truth differential results and compared them to our inferred results (Methods). Considering the first corrupted dataset, although no expression data were observed in the B cell population, we found high concordance between the observed and predicted log fold-change (log_FC_) values (Fig. 5a, Pearson’s correlation 0.57). When examining genes that are preferentially expressed in B cells (observed log_FC_ > 1) this became more evident (Pearson’s correlation 0.74). Similarly, with the second corrupted dataset, we found high concordance between observed and predicted differences of accessibility (Fig. 5b, Pearson correlation 0.67).

Among the top most differentially expressed genes detected with the imputed expression values, we found known B cell markers, including *IGLC3*, *IGHM*, *CD79A* and *IGHD* (Supplementary Table 1 and Fig. 5c). Another example for a differentially expressed gene is *CR2*, a membrane protein that is normally expressed by both B and T cells, was predicted specifically in the corresponding compartments (Fig. 5d). Overall, we identified 1621 significantly differential genes (false discovery rate (FDR) of less than 0.05), of which 75% were also identified with the held-out data at a 5% FDR, a modest but significant enrichment (odds-ratio 1.22, hypergeometric test *P* < 1.9 × 10^−35^; Supplementary Table 1). Increasing the threshold of significance (on the FDR for the standard analysis and the Bayes factor for the MultiVI results) increased the overlap between the sets of results indicating that the results are more consistent for more highly significant genes (Fig. 5e). Similarly, we identified 922 differentially accessible regions (FDR of^22^ 0.05, Supplementary Table 2), of which 86% were also identified with the held-out data at 5% FDR (odds-ratio 1.57, *P* < 1.7 × 10^−95^). As in the gene expression analysis, the overlap between the inferred and observed differential accessibility analyses increased with the significance thresholds (Fig. 5f).

Taken together, these results demonstrate that MultiVI can be used to impute missing modalities even for populations that were only identified in a single-modality dataset. This unlocks the ability to leverage multimodal data to reanalyze existing single-modality datasets and impute the missing modality: chromatin landscape for existing scRNA experiments, and gene expression for existing scATAC experiments, as well as performing differential analyses using these imputed values.

### MultiVI models three modalities and enables data imputation

**Fig. 6:**
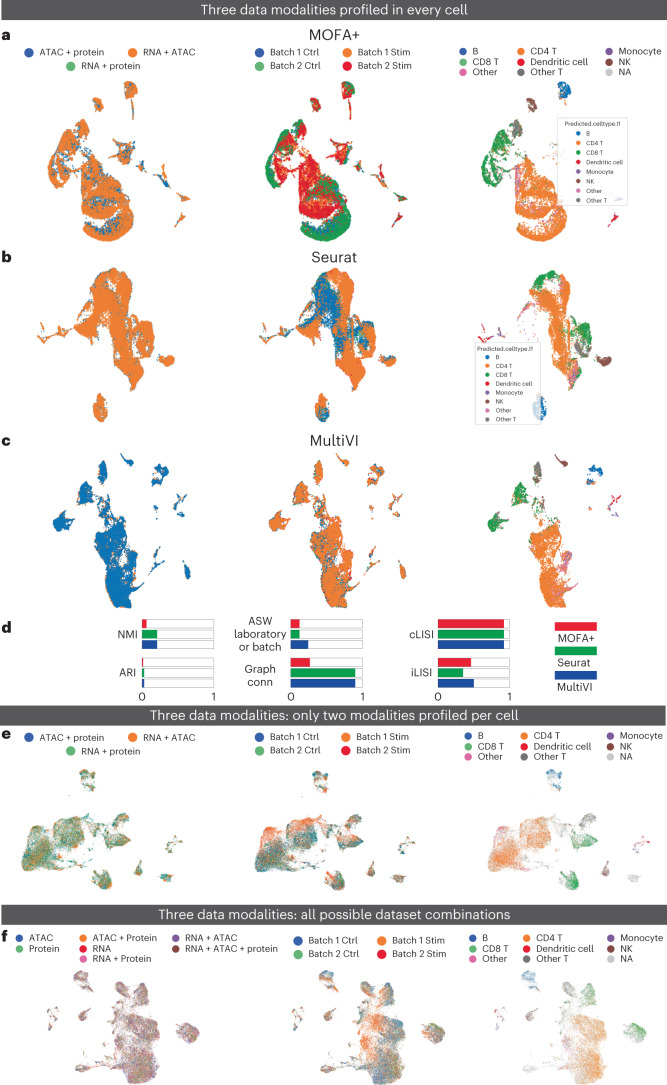
MultiVI integrates transcriptional, chromatin accessibility and protein expression information into a meaningful latent space. **a**–**c**, UMAP representations computed for a dataset of PBMCs collected using the DOGMA-seq protocol by MOFA (**a**), Seurat WNN (**b**) and MultiVI (**c**). In each panel, cells are color coded by replicate (left), condition (middle) and cell type (right). **d**, Summary metrics describing batch correction and biological preservation color coded by method. **e**, PBMC DOGMA-seq dataset in which no cells has three modality information. UMAP representation of the latent representation computed by MultiVI in which cells are color labeled by their modality, batch and cell type. **f**, PBMC DOGMA-seq dataset in which cells are measured in every possible combination. UMAP representation of the latent representation computed by MultiVI in which cells are color labeled by their modality, batch and cell type. Stim, stimulation; Ctrl, control; ASW, average silhouette width; NMI, normalized mutual information; Graph conn., graph connectivity.

To test the ability of MultiVI to integrate more than two modalities, we added measurements of protein abundance on the cell surface (with CITE-seq) and accounted for these in the MultiVI model using distributional assumptions similar to TotalVI^16^ (Methods). To assess the ability of MultiVI to create meaningful tri-modal latent representations, we used the two PBMC datasets profiled with DOGMA-seq^7^ and TEA-seq^6^ protocols. For each dataset separately we then integrated the different samples (Fig. 6a–c and Supplementary Fig. 9a–c), and evaluated the results using batch correction and biological-conservation metrics. As benchmark, we compared MultiVI to MOFA+ (ref. ^9^) and Seurat WNN^8^ (notably, the latter was designed for the case when all samples have all modalities). In both datasets, the latent space inferred by MultiVI performed on par with the two benchmark methods (Fig. 6d and Supplementary Fig. 9d). Next, we explored MultiVI’s ability to integrate tri-modal paired and unpaired samples. To benchmark this regime, we used the DOGMA-seq dataset to artificially create cells that are only profiled in two of the three modalities (RNA + chromatin, chromatin + protein or RNA + protein) (Fig. 6e). Overall we observed that MultiVI’s latent representation effectively removes batch effects and preserves cell-type identity. We quantified this performance by computing integration metrics and comparing against a principal components analysis (PCA) latent space. PCA reseaches a better adjusted Rand index (ARI) score, while MultiVI outperforms in terms of batch connectivity (iLISI), graph connectivity and all silhouette-based metrics (Methods and Supplementary Fig. 9e).

Next, we examined how MultiVI handles a complex experimental design in which cells are profiled in all possible combinations of modalities. We split cells in the DOGMA-seq dataset, selectively removed information from different modalities and created datasets that have only one (out of three) modality or two (out of three) modalities. We found that the resulting integrated space (Fig. 6f) ameliorates batch and technologies effects while maintaining cell-type biological information, as in previous scenarios. Again, we assessed MultiVI’s performance by comparison to PCA embedding computed on the raw data and showed superior integration performance (Supplementary Fig. 9e). Overall, these results demonstrate that MultiVI is able to ameliorate batch effects while preserving biological heterogeneity, even in the complex scenario in which three data modalities are present at different combinations.

Last, we assessed the performance of MultiVI to impute missing data in this tri-modal setting. We artificially unpaired 75% of the data, such that each cell is represented by three copies with only RNA, chromatin accessibility or protein expression data (resulting in a dataset in which 8% of cells have all tree modalities and the rest have only one of the three modalities). We find that the imputation of the missing modalities, generated by MultiVI (Supplementary Fig. 10) correspond to the observed values. These include Spearman correlations of 0.78 in the gene expression values, independent of the single modality used as input data (using smoothed observations, as above). We observed a similar outcome in the case of chromatin accessibility, with a Spearman correlation of 0.73 and 0.76 between the smoothed observed values and the imputed ones when only RNA or protein information is available, respectively. Our model for the protein data was designed to control for the nonnegligible background component in the signal (which may result of nonspecific binding of antibodies). We therefore first calculated the foreground (‘denoised’) component of all observed protein expression values using TotalVI (ref. ^16^). Since the protein imputed values in MultiVI are also generated with a similar two component model, we were able to compare the imputed foreground signals to foreground signals that were inferred from the respective hidden observations. We find that the imputed values are also correlated with the observed data (with Spearman correlations of 0.53 when only chromatin accessibility data or gene expression data are available).

In summary, the inclusion of protein data into our analysis highlights the ability of MultiVI to handle additional data types and leverage them for a joint analysis with measurements of chromatin accessibility and gene expression.

## Discussion

MultiVI is a deep generative model for integrated analysis of multimodal and single-modality single-cell datasets. MultiVI uses jointly profiled data to learn a multimodal model of data sources and to relate individual modalities on the same population of cells. The model accounts for technical sources of measurment noise and can correct for additional sources of unwanted variation (for example, batch effects). MultiVI learns a rich embedding of the data coalescing information present in each individual data type, which can be used for downstream analyses.

Multiomic integration algorithms have been recently classified based on their ability to infer latent representations from shared cells across measurements (vertical integration scenario), shared features across datasets (horizontal integration scenario)^23,24^ or subsets of any of the previous (mosaic integration scenario). MultiVI could be considered a vertical integration scheme, when purely multiomic data are analyzed and different modalities are measured in the same cells. At the same time, MultiVI’s ability to integrate transcriptional and protein data, when only a subset of genes are shared across technologies, placed it within the horizontal integration scenario. Last, MultiVI’s capacity for integrating multiple modalities when only a subset of cells is shared across modalities also qualifies it as a mosaic algorithm. These characteristics highlight MultiVI’s ability to work under many scenarios, in contrast to previous algorithms that are optimized to work just in one condition^25,26^.

Recent algorithms for the analysis of multimodal data were developed to process paired datasets, in which both modalities have been profiled at the same cell^9,10^. Most algorithms, however, handle multimodal data, but lack the ability to integrate single-modality datasets. While this task is possible to achieve with the Seurat code base^8^, the respective methods we used here were not specifically designed to this end, and their performance was not tested for this task. Here, we have shown that use of deep generative modeling can effectively combine unpaired scRNA and scATAC data with multimodal single-cell data, generating a meaningful low-dimensional representation of the cells’ state that captures information about both their transcriptome and epigenome. This joint representation is achievable even when the amount of paired data is minimal, thus opening exciting opportunities for future studies in which only a small amount of paired data can be sufficient for deriving a more nuanced interpretation of single-modality data. In contrast to Cobolt, or other two-modality algorithms^27,28^, we demonstrate that MultiVI is able to integrate information from additional molecular properties of cells, such as surface protein expression. However, MultiVI might be limited in its ability to analyze datasets with a small number of cells, both due to the scale of data required to train neural networks, as well as the necessary amount of information needed to correctly learn multiple modelity-specific embeddings.

An additional key capability that is unique to MultiVI is the inference of the missing modality. We have demonstrated that we can identify differential gene expression in subpopulations for which only chromatin accessibility data are available and distinguishing chromatin features for subpopulations for which only gene expression data are available. These results open the way for exciting future applications: first, MultiVI and similar methods have the potential to enable a reanalysis of the large compendia of available single-modality datasets with a relatively small amount of additional paired data, thus potentially leading to more comprehensive characterizations of cell state. Second, it can facilitate cost-effective designs for future studies, in which only a subset of samples need to be profiled with the (more costly) multimodal protocol. Last, MultiVI expedites the transfer of information and analysis results across modalities, such as the case in which RNA velocity could be ported to cells in which only chromatin information has been profiled.

In summary, MultiVI is able to seamlessly integrate single- and multimodal data, process information from different laboratories or technologies and create a rich joint representation (low and high dimensional) that harnesses all available information. It is implemented in the scvi-tools framework^29^, making it easy to configure, train and use.

## Methods

### The MultiVI model

MultiVI inherits generative models describing chromatin accessibility and transcriptional observations from scVI (ref. ^14^), peakVI (ref. ^15^) and TotalVI (ref. ^16^). Briefly, let ${X}_{\mathrm{R}}\in {{\mathbb{N}}}_{0}^{C\times G}$ be a scRNA-seq genes-by-cell matrix with *C* cells and *G* genes, where ${x}_{\mathrm{R}}^{cg}\in {{\mathbb{N}}}_{0}$ is the number of reads from cell *c* that map to gene *g*. Let ${X}_{\mathrm{A}}\in {{\mathbb{N}}}_{0}^{C\times J}$ be a scATAC-seq region-by-cell matrix with *C* cells and *J* regions, where ${x}_{A}^{cj}\in {{\mathbb{N}}}_{0}$ is the number of fragments from cell *c* that map to region *j*. Let ${X}_{P}\in {{\mathbb{N}}}_{0}^{C\times P}$ be a protein-by-cell matrix with *C* cells and *P* proteins, where ${x}_{P}^{cp}\in {{\mathbb{N}}}_{0}$ is the number of fragments from cell *c* that map to protein *g*.

MultiVI models the probability of observing *x*_*cj*_ counts in a gene by using a negative binomial distribution,

1 $${x}_{\mathrm{R}}^{* } \approx {{{\rm{NegBin}}}}\left({\ell }_{c}{\rho }_{cg},{\theta }_{g}\right)$$

where *ℓ*_*c*_ is a scaling factor that captures cell-specific biases (for example, library size), *ρ*_*cg*_ is a normalized gene frequency and *θ*_*g*_ models the per gene dispersion. The probability of observing a region as accessible is modeled with a Bernoulli distribution,

2 $${x}_{\mathrm{A}}^{* } \approx {{{\rm{Ber}}}}\left({\ell }_{c}{p}_{cj}{r}_{j}\right)$$

where *p*_*cj*_ captures the true biological heterogeneity and *r*_*j*_ captures region-specific biases (for example, width, sequence). Last, MultiVI models protein expression with a mixture of negative binomial distributions that encompass background and foreground protein expression:

3 $${x}_{\mathrm{P}}^{* } \approx {\pi }_{1}{{{\rm{NegBin}}}}\left({\ell }_{c}{\beta }_{cg}^{b},{\theta }_{g}^{b}\right)+(1-{\pi }_{1}){{{\rm{NegBin}}}}\left({\ell }_{c}{\alpha }_{cg}^{\,f}{\beta }_{cg}^{b},{\theta }_{g}^{\,f}\right)$$

In this model, *π*_1_ accounts for the mixture proportion, *β* for the background expression level and *α* ≥ 0 is a value that corrects for foreground expression. In the observational models, the normalized gene frequency per cell, the normalized peak accessibility and the background expression level are inferred from data using deep neural networks. The scaling factor, the region-specific and the per gene dispersion parameters are optimized directly (this is in contrast to the original implementation of scVI in which library size was modeled using a lognormal distribution).

Next, for each cell, normalized gene frequencies *ρ*_*cg*_*,* accessibility biological heterogeneity *p*_*cj*_ and background and foreground protein expression ${\alpha }_{cg}^{\,f}$ and ${\beta }_{cg}^{b}$, are estimated using a latent representation as in VAE^13^. Briefly, each modality is assign their own latent representation, a isotropic multivariate normal distribution ${Z}_{c}^{\,\mathrm{A}} \approx {{{\rm{MVN}}}}(0,1)$, ${Z}_{c}^{\,\mathrm{R}} \approx {{{\rm{MVN}}}}(0,1)$ and ${Z}_{c}^{\,\mathrm{P}} \approx {{{\rm{MVN}}}}(0,1)$. Then, with the purpose of bringing all representations together, they are combined by taking their average (for example, in the case of two modalities profiled such as ATAC and RNA, we have ${Z}_{c}=\frac{{Z}_{c}^{\,\mathrm{A}}+{Z}_{c}^{\,\mathrm{R}}}{2}$). This merged representation is then used to decode all model parameters.

We explore alternative modality weighting schemes. Our default mode involves an average across modality latent representations, termed ‘equal’ in our software release and in the Supplementary Information (example for two modalities ${Z}_{c}=\frac{{Z}_{c}^{\,\mathrm{A}}+{Z}_{c}^{\,\mathrm{R}}}{2}$). Alternatively, a global weighted average scheme equal across all cells, *w*_m_, such that ∑_modalities_*w*_m_ = 1 (example for two modalities ${Z}_{c}={w}_{\mathrm{A}}{Z}_{c}^{\,\mathrm{A}}+{w}_{\mathrm{R}}{Z}_{c}^{\,\mathrm{R}}$) . Last, a cell-specific weight across modalities, *w*_m,c_, such that ∑_modalities_*w*_m,c_ = 1 (example for two modalities ${Z}_{c}={w}_{{\mathrm{A}},c}{Z}_{c}^{\,\mathrm{A}}+w+{\mathrm{R}},c{Z}_{c}^{\,\mathrm{R}}$). In both cases, the weights are learnable parameters optimized along with the rest of the model.

### MultiVI inference model

We use variational inference^30^ to compute posterior estimates of model parameters using the following variational approximation:

4 $$\begin{array}{ll}q({z}^{\mathrm{R}},{z}^{\mathrm{A}},{z}^{\mathrm{P}},r,\ell ,\theta | {x}_{\mathrm{A}},{x}_{\mathrm{R}},{x}_{\mathrm{P}})&=q({z}^{\mathrm{R}}| {X}_{\mathrm{R}})\cdot q({z}^{A}| {X}_{\mathrm{A}})\\ \cdot q({z}^{\mathrm{P}}| {X}_{\mathrm{P}}){\delta }_{{\ell }}{\delta }_{{\theta }}{\delta }_{{\theta }^{g}}{\delta }_{{\theta }^{f}}{\delta }_{{r}},{\delta }_{{\pi }_{1}}\end{array}$$

where the delta distribution, *δ*, highlights the fact that parameters *ℓ*, *r*, *θ* and *π*_1_ are inferred from the data as point estimates and optimize directly. The cell-specific factor *ℓ*_*c*_ ∈ *ℓ* is computed from the input data for cell *c* via a deep neural network ${f}_{\ell }:{{\mathbb{N}}}_{0}^{C}\to \left[0,1\right]$. The region-specific factor *r*_*j*_ ∈ *r*, since it is optimized across samples, is stored as a *J*-dimensional tensor, used and optimized directly. In the case of each latent representation, encoders are computed as ${h}_{z}^{\mathrm{Transc}}:{{\mathbb{N}}}_{0}^{C}\to \left({{\mathbb{R}}}^{D},{{\mathbb{R}}}^{D}\right)$, ${h}_{z}^{{\mathrm{Chrom}}}:{{\mathbb{N}}}_{0}^{C}\to \left({{\mathbb{R}}}^{D},{{\mathbb{R}}}^{D}\right)$, ${h}_{z}^{\mathrm{Protein}}:{{\mathbb{N}}}_{0}^{C}\to \left({{\mathbb{R}}}^{D},{{\mathbb{R}}}^{D}\right)$ where each of them computes the distributional parameters of a *D*-dimensional multivariate normal random variable: ${z}_{c}^{\mathrm{Modality}} \approx {\mathrm{MVN}}\left(\mu ={[{h}_{z}^{\mathrm{Modality}}\left({x}_{c}\right)]}_{1},{\sigma }^{2}={[{h}_{z}^{\mathrm{Modality}}\left({x}_{c}\right)]}_{2}\right)$. The subscripts 1 and 2 in the last equation reflect the fact that each encoder computes both the mean and the variance of the distribution.

Using the variational approximation, the evidence lower bound is computed and optimized with respect to the variational and model parameters using stochastic gradients. To enforce the similarity between chromatin and transcription latent representations, we add to the evidence lower bound a term that penalizes the distance between representations using a symmetric Jeffrey’s divergence between distributions $$d\,({Z}_{c}^{\,\mathrm{A}},{Z}_{c}^{\,\mathrm{R}})={{{\rm{symmKL}}}}(q({z}_{c}^{\mathrm{A}}),q({z}_{c}^{\mathrm{R}}))={{{\rm{KL}}}}(q({z}_{c}^{\mathrm{A}}),q({z}_{c}^{\mathrm{R}}))+{{{\rm{KL}}}}(q({z}_{c}^{\mathrm{R}}),q({z}_{c}^{\mathrm{A}}))$$. In the case of three or more distributions, we extend the penalty to match every possible set of distributions (when we include protein data, $$d({Z}_{c}^{\,\mathrm{A}},{Z}_{c}^{\,\mathrm{R}},{Z}_{c}^{\,\mathrm{P}})={{{\rm{symmKL}}}}\left(q({z}_{c}^{\mathrm{R}})\right.,q({z}_{c}^{\mathrm{A}})+{{{\rm{symmKL}}}}\left(q({z}_{c}^{\mathrm{R}})\right.,q({z}_{c}^{\mathrm{P}})+$$$${{{\rm{symmKL}}}}(q({z}_{c}^{\mathrm{A}}),q({z}_{c}^{\mathrm{P}}))$$. Last, we explored an alternative penalization scheme in which we replace the symmetric KL divergence by an MMD penalty (MMD^18^). Additional information about the model definition and inference procedure can be found at https://docs.scvi-tools.org/en/stable/user_guide/models/multivi.html.

### Training procedure

By default, MultiVI is optimized using AdamW^31^ with a learning rate of 0.0001, weight decay of 0.001 and minibatch size of 128. As in previous models, we trained on 90% the data and used 10% as a validation set. We selected an initial training plan consisting of 500 epochs but the model is stopped early if there is no improvement in the reconstruction loss on the validation dataset for 50 epochs (early stopping). We down-weighted the KL divergence between the latent representation and its prior during the first 50 epochs (for *i* ∈ [1, 50], ${{{\rm{KL}}}} \cdot \frac{i}{50}$). In addition, a domain adaptation penalty is included in the training schemes to increase mixing in the latent space^32,33^. Briefly, a classifier is created using a two-layer feed forward neural network with 32 hidden units. Its output is the probability for each cell to belong to one of the batch keys. We use the output of this classifier to create a cross-entropy loss that is adversarially trained.

### Modeling differences between MultiVI and Cobolt

While conceptually similar, MultiVI and Cobolt have several key differences in design and implementation choices. MultiVI offers additional functionalities due to its generative model, that is denoising, imputation, uncertainty estimation and differential analyses, which are discussed in detail in this paper. In addition to those, we detail several other differences between the methods: (1) MultiVI uses a distributional average and penalization to mix the latent representations, compared with the classical product of experts calculation used by Cobolt. (2) The distributional assumptions made by the models are different: MultiVI uses tailored noise models for each modality (negative binomial for expression, Bernoulli for accessibility) and uses a deep neural network for the generative component of the model as well as the inference component. In contrast, Cobolt uses a multinomial likelihood for both modalities and uses a linear transformation as a generative model. (3) MultiVI explicitly avoids overfitting the data, in both the architecture (for example, dropout layers) and training procedure (holding out data to use for early-stop if the model overfits), whereas Cobolt does not contain such guardrails.

### Benchmarking and evaluation

#### Dataset preprocessing

The 10X multiomic unsorted PBMC dataset was downloaded from the company website. For artificial unpairing analyses, the processed peak-by-cell matrix was downloaded and filtered to remove features that are detected in fewer than 1% of the cells. For the mixed-source PBMC dataset, the fragment file was downloaded and reprocessed using CellRanger-ARC (v.2.0.0) with the Satpathy hg38 peaks. The Satpathy dataset was downloaded from the Gene Expression Omnibus (GEO) (accession no. GSE129785); specifically the processed peak-by-cell matrix and metadata files: scATAC-Hematopoiesis-All.cell-barcodes.txt.gz, scATAC-Hematopoiesis-All.mtx.gz and scATAC-Hematopoiesis-All.peaks.txt.gz. We then filtered the data to only include peaks that were detected in at least 0.1% of the data, and lifted those peaks over from the hg19 to the hg38 genome reference using the UCSC liftover utility^34^. The Ding dataset was downloaded from GEO (accession no. GSE132044); specifically the pbmc data: pbmc_hg38_count_matrix.mtx.gz, pbmc_hg38_cell.tsv.gz and pbmc_hg38_gene.tsv.gz.

Matching cell-type annotation was downloaded from SCP (accession no. SCP424). After preprocessing, the reanalyzed 10X dataset was combined with both single-modality datasets and the features were filtered to remove features (either genes or peaks) that were detected in fewer than 1% of the cells.

The DOGMA-seq dataset^7^, containing paired scRNA, scATAC and surface protein abundance observations were downloaded from GEO (accession no. GSE156478); specifically the four samples containing all three modalities: CD28_CD3_*. The ATAC observations were merged using ArchR^35^ using default arguments to produce a unified set of peaks called from all four samples. For model training, we only used features that were detected in at least 1% of cells. For the analyses included in this paper, only cells originating from the DIG_CTRL sample were used.

#### RNA-based Seurat integration

This integration modality disregards multiomic information and only RNA information is considered from multiome cells. Briefly, RNA information is first integrated and then chromatin accessibility is integrated using gene activity scores (RNA-based method) or RNA imputed values (RNA-based imputed method).

In more detail, cells were separated into three different datasets, multiomic cells (using only expression data), RNA-only cells and ATAC-only cells. Seurat objects were created for multiome and RNA-only data, and were then normalized, scaled and the first 50 principal components are calculated. For ATAC-only cells, a Seurat object was created, gene activity scores were calculated, scaled and principal components were computed. To integrate the three datasets, integration anchors (using FindIntegrationAnchors) were calculated and the data were then integrated (using IntegrateData). The RNA-based method uses gene activity scores as representative values from the ARAC-only cells. The RNA-based imputed method includes an additional step in which RNA imputed values are calculated from gene activity scores by running FindTransferAnchors and TransferData. In this integration method, RNA imputed values are used as representative values from ATAC-only cells. Finally, integrated data were then scaled and principal components were calculated to generate the final latent space. Across these integration methods, we followed the standard recommended procedure for analyzing data with Seurat given in their tutorials^36^.

#### WNN-based Seurat integration

This approach aims to leverage information from both modalities (chromatin accessibility and expression values), using the newly described WNN approach from Seurat V4 (ref. ^8^). We first created a WNN graph using multiomic information and then project chromatin and transcriptional information onto this.

We begin by separating cells in unpaired datasets into three different datasets, multiomic cells (with both expression and chromatin data), RNA-only and ATAC only. First, multiome latent representation is found by using the *sctransform* function and principal components on the expression data and latent semantic analysis (LSA) (TF-IDF decomposition followed by singular value decomposition) on the chromatin data. Next, multimodal neighbors and the first 50 supervised PCA are calculated. To merge RNA- and ATAC-only data to multiome representation, transfer anchors (FindTransferAnchors) are computed on RNA-only data and gene activity scores on ATAC only and each dataset is integrated using IntegrateEmbeddings function. Finally, datasets and dimensionality reductions are merged and uniform manifold approximation and projection (UMAP) is visualized using the merged information.

#### Neighbor rank distance calculation

For artificially unpaired cells, each cell has two unpaired representations in the latent space. Given cell *c* with representations *c*_a_ and *c*_b_, let $S\left({c}_{\mathrm{a}},K\right)$ be the set of KNN to *c*_a_. We then define $\delta \left({c}_{\mathrm{a}},{c}_{\mathrm{b}}\right)$ as the minimal *K* for which cell *c*_b_ is among the KNN of cell *c*_a_, $\min \left\{k:{c}_{\mathrm{b}}\in S\left({c}_{\mathrm{a}},k\right)\right\}$.

#### LISI score calculation

Enrichment scores were computed as they were in our previous work^15^, and similarly to the LISI scores described in the Harmony paper^17^. Briefly, given a latent representation *R*, an integer *k* and the modality labels (expression, or accessibility) *L*, we compute *G*_*R*,*k*_ the KNN graph from *R* with *k* neighbors. Using *G*_*R*,*k*_, we compute for each cell the proportion of neighbors that share the same modality: ${s}_{\mathrm{i}}=\frac{1}{k}{\sum }_{j\in {G}_{R,k}(i)}{\mathbb{1}}\left({L}_{i}={L}_{j}\right)$. The enrichment score is the average score across all cells, $\bar{s}$, normalized by the expected score for a random sample from the distribution of labels: $E\left[s\right]={\sum }_{\ell \in \{L\}}{p}_{\ell }^{2}$, with *p*_*ℓ*_ being the proportion of each modality.

#### Extended integration metrics

We computed extended integration metrics using the scib^19^ package. Briefly, to quantify integration throughout the paper, we computed the ARI, normalized mutual information, graph connectivity, batch LISI (iLISI), cell-type LISI (cLISI), kBET, silhouette width (label silhouette) and the average silhouette width as proposed previously. To quantify metrics depending on clustering of the data, we first ran the provided functions to optimized clustering resolution. We provided cell-type labels as labelkey and corresponding label under evaluation as batchkey.

#### Estimating imputation uncertainty

We estimated the uncertainty of the model for each imputed value by sampling from the latent space (*n* = 15). Next, for each imputed feature (gene or loci), we computed the mean and standard deviation. More consistent predictions corresponded to less uncertainty.

#### KNN-based estimate of accessibility

To estimate accessibility without using MultiVI, we computed a lower dimensional representation of the data using LSA (top 30 components), then for each cell we computed the average accessibility profile of the 50 nearest neighbors in the LSA space. This created a smooth estimate of accessibility using highly similar cells, mitigating the effect of false observations.

#### Expression smoothing

Expression smoothing was achieved by taking the top 30 principal components of the expression data (computed with PCA), computing the KNN graph (for *K* = 50) and averaging the expression values of the neighbors for each cell (scaled by library size).

#### Differential analyses with held-out data

To identify a distinct population of cells, we used the Leiden community detection algorithm^37^, then examined the expression levels of known marker genes (CD79A, CD19) to identify the cluster of B cells. We then unpaired the data within the cluster, once by removing all expression data from the B cells and once by removing all accessibility data from the clusters. Since the data were already unpaired, this resulted in several cells with no observations at all and those were removed from the dataset.

#### Differential expression using held-out data

Differential expression was computed in two ways: (1) using the held-out data, values were normalized per cell by dividing the expression levels by the total number of reads in the cell. The log_FC_ values were then computed by dividing the mean expression values in the two groups. Statistical significance was determined using Wilcoxon rank-sum test. (2) Without the held-out data, using MultiVI, in a procedure described by Lopez et al.^14^, in which samples from the latent space and uses the generative model to estimate expression profiles. Statistical significance was then determined using Bayes factors, as well as an FDR approach described by Lopez et al.^22^.

#### Differential accessibility using held-out data

Differential accessibility was computed equivalently to differential expression. (1) Using held-out data, values were normalized using the TF-IDF transformation, differential accessibility was computed by subtracting the mean accessibility in the reference group from the same value in the target group. Statistical significance was determined using Wilcoxon rank-sum test. (2) Without the held-out data, using MultiVI, using the procedures described in our previous work^14,15,22^.

### Reporting summary

Further information on research design is available in the Nature Portfolio Reporting Summary linked to this article.

## Online content

Any methods, additional references, Nature Portfolio reporting summaries, source data, extended data, supplementary information, acknowledgements, peer review information; details of author contributions and competing interests; and statements of data and code availability are available at 10.1038/s41592-023-01909-9.

## Supplementary information

Supplementary Information Supplementary Figs. 1–10.

Reporting Summary

Supplementary Table 1 Supplementary tables 1 and 2.

## Data Availability

All data used in this paper are publicly available via the original publications and releases. TEA-seq datasets were download from GEO, accession number GSE158013. DOGMA-seq datasets were downloaded from GEO accession no. GSE156478. The Satpathy scATAC-seq dataset was downloaded from GEO, accession no. GSE129785. The Ding scRNA-seq dataset was downloaded from GEO accession no. GSE132044. The 10X PBMC sample dataset is available from the company website: https://www.10xgenomics.com/resources/datasets

## Code availability

The code for MultiVI is publicly available via the scvi-tools suite at scvi-tools.org. Intermediate data, trained models used in this paper and the custom notebooks needed to generate the figures in this paper are all posted and available on Zenodo^38^.
