# Supplementary material for: MultiVI: deep generative model for the integration of multimodal data
Source: Nat Methods. 2023 Jun 29;20(8):1222–31. doi: 10.1038/s41592-023-01909-9 (PMC10406609; doi:10.1038/s41592-023-01909-9)
Supplement: Supplementary file 1 — Supplementary Figs. 1–10. [file 41592_2023_1909_MOESM1_ESM.pdf]

nature methods

Article

https://doi.org/10.1038/s41592-023-01909-9

MultiVI: deep generative model for the integration of multimodal data

In the format provided by the authors and unedited

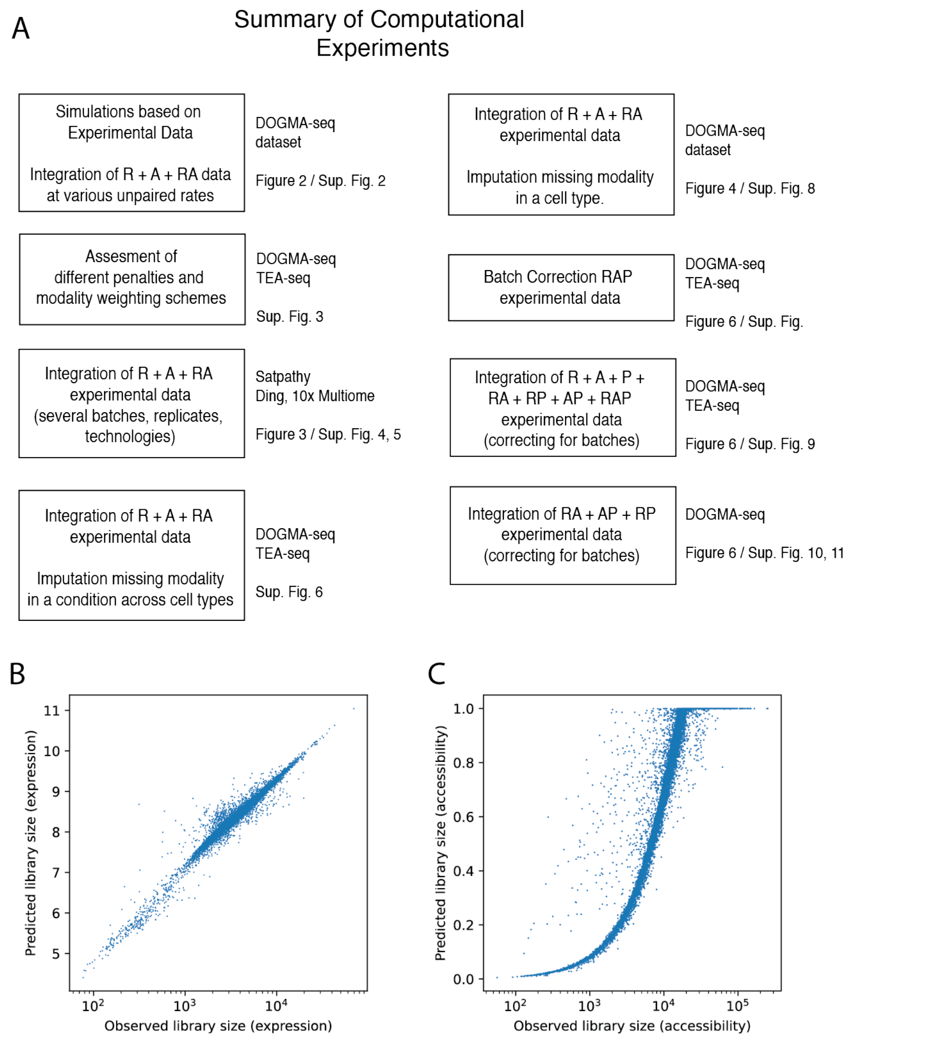

Figure S1: **Summary of Computational Experiments and Library Size Experiments**. A)Summary of Computational Experiments Performed in the Manuscript. In each box we describe the type of experiment performed. Next to each box we describe the datasets used in the calculation and the figure in which results are displayed. B-C) Comparison of observed and predicted library sizes for RNA (B) and ATAC (C)

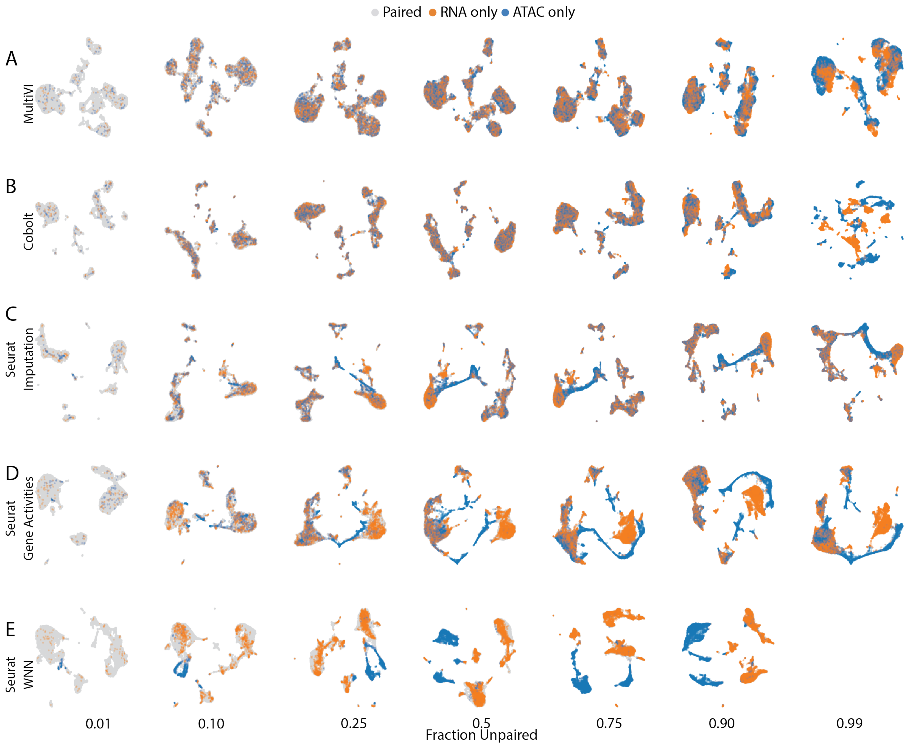

Figure S2: **Extended Integration results depicting mixing of cells in data sets with different fraction of cells unpaired.** UMAPS of latent representations for MultiVI (A), Cobolt (B), Seurat imputation method (C), Seurat Gene Activity Scores method (D), and Seurat wKNN method (E).

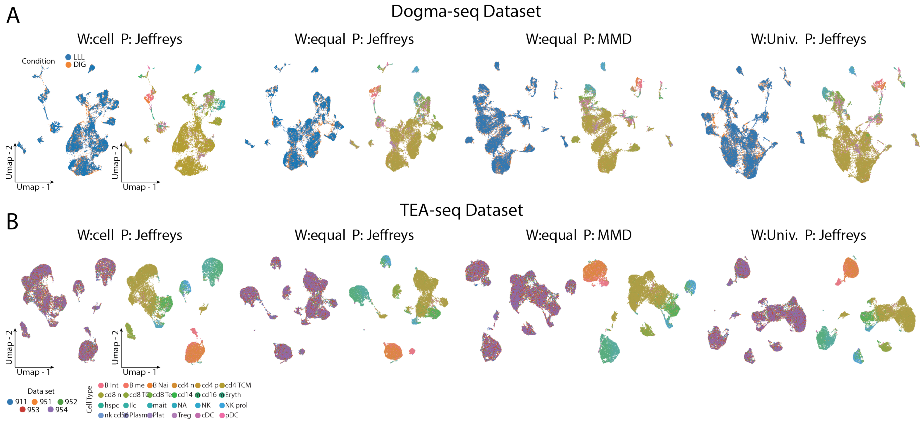

C

TEA-seq Dataset

	W:equal P: MMD	W:equal P: Jeffreys	W:cell P: Jeffreys	W:Univ. P: Jeffreys
ari	0.430	0.416	0.410	0.411
nmi	0.629	0.618	0.618	0.613
clisi	0.994	0.994	0.994	0.994
ilisi	0.700	0.690	0.686	0.700
graph_conn	0.770	0.760	0.771	0.787
kBET	0.634	0.654	0.597	0.646
ASW_label	0.285	0.279	0.275	0.272

Dogma-seq Dataset

	W:equal P: MMD	W:equal P: Jeffreys	W:cell P: Jeffreys	W:Univ. P: Jeffreys
ari	0.150	0.292	0.545	0.287
nmi	0.383	0.388	0.421	0.382
clisi	0.987	0.986	0.986	0.986
ilisi	0.827	0.783	0.791	0.789
graph_conn	0.822	0.812	0.81	0.798
kBET	0.630	0.559	0.531	0.556
ASW_label	0.183	0.215	0.222	0.212

Figure S3: **MultiVI analysis of different modality weighting schemes and penalizations.** We assess different modeling assumptions in two PBMC datasets, DOGMA-seq and TEA-seq. A, B) MultiVI Latent Representation integrating DOGMA-seq (A) and TEA-seq datasets (B). In every panel, conditions or batches are color-coded on the left and annotated cell types on the right. C) Integration metrics for each integration scheme tested. W: weighting scheme across modalities. Modality-specific weight per cell (termed cell), average across modalities (equal) or inferred modality weight, constant across cells (universal). P: penalty imposed across modalities' latent representation, Jeffreys or MMD penalties. Metrics computed in panel C) are explained in supplemental methods.

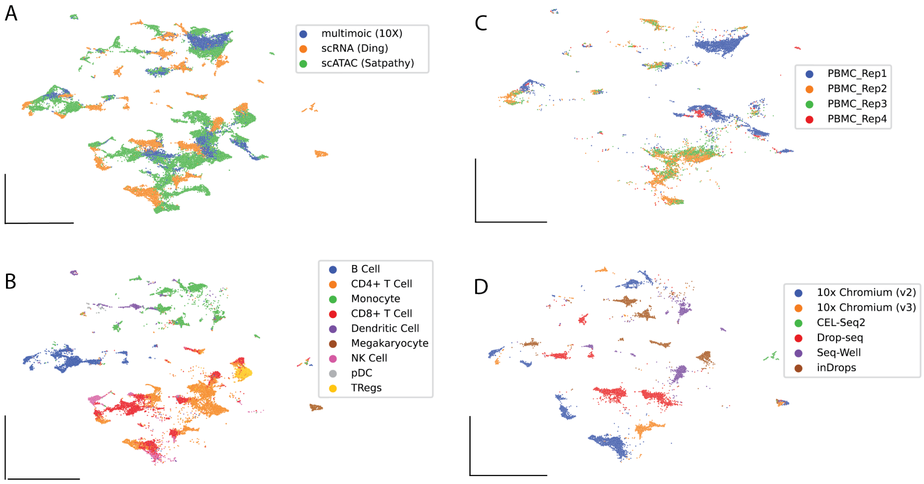

Figure S4: **Latent representation of mixed sources data sets (Fig. 2) in which no batch correction techniques have been applied.** We integrated three PBMC datasets in which only multi-modal data (10X multiome), only ATAC-seq information (Satpathy et al) and only RNA-seq information (Ding et al) is present without correcting for batch or modalities effects. A)-D) UMAP representation computed from the latent space of MultiVI in which cells are color labeled by their dataset (A), their cell type (B) or ATAC-seq cells are labelled by the replicate in which they were collected (C) or RNA-seq cells are labelled by their collection experimental technology.

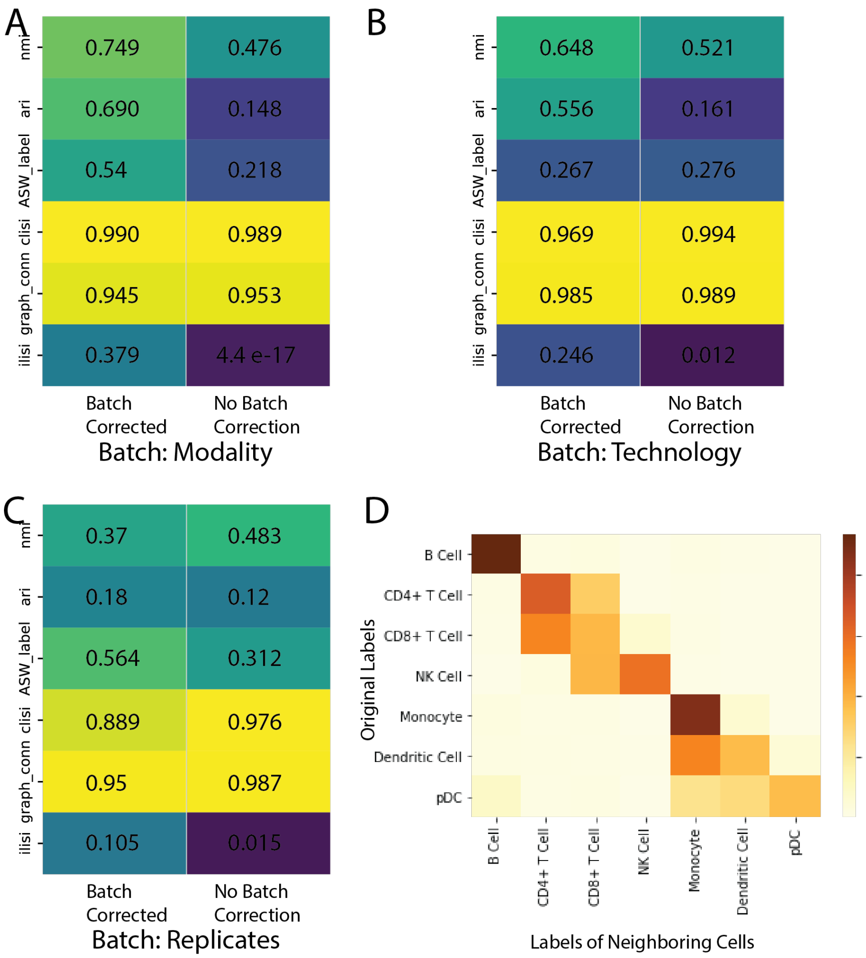

Figure S5: **Integration metrics for mixed sources data sets (Fig. 2).** Metrics evaluating mixing of A) modalities, B) technologies, and C) replicates. Metrics like nmi, ari, clisi and graph connectivity evaluate cell type information preservation while ASW, and ilisi evaluate batch correction (supplemental methods). D) Distribution of labels of cross-modal neighbors. For each single-modality cell, we looked at the cell-type labels of the 50 nearest neighbors of the other modality. We then visualize the distribution of neighbor-labels, aggregated by the label of the original cell. For example, almost 100% of cross-modality neighbors of B-cells are also labelled B-cells

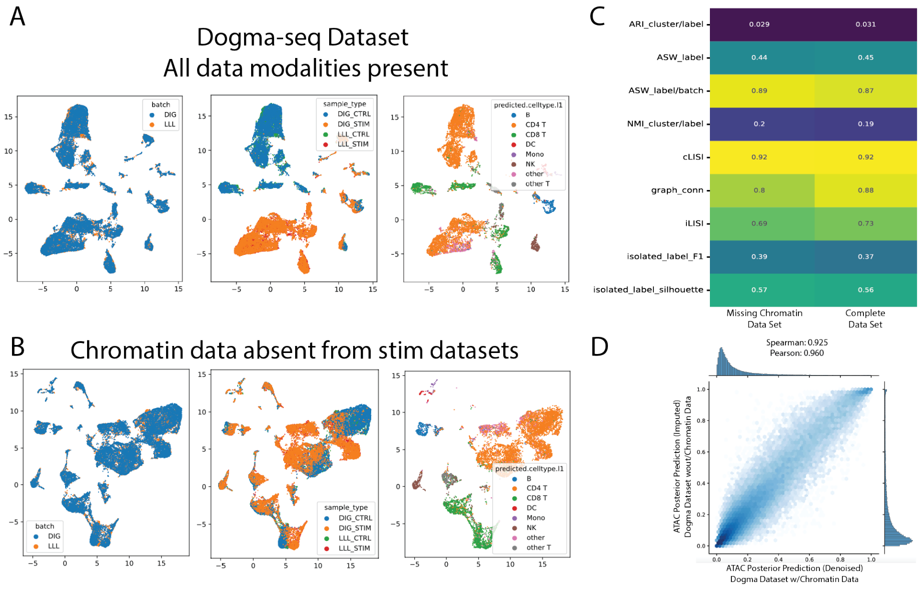

Figure S6: **MultiVI accurately imputes missing data when single-modality information is missing in cells arising from a perturbed condition.** A) reproduction of UMAP visualization for a 10x PBMC multiome DOGMA-seq data set in which all modalities are present in each cell. B) UMAP visualization for the same data set in which chromatin information has been removed in all stimulated cells for all batches in the experiment. In panel A) and B), cells are color coded by batch (left), condition (middle) and cell type (right). C) summary metrics showcasing similar latent space integration performance in both scenarios. D) We imputed chromatin information for stimulated cells when such information is absent during the training procedure (B), and compare it against its denoised estimates (A). The high correlation reveals among both sets of values reveals accurate performance in this setting.

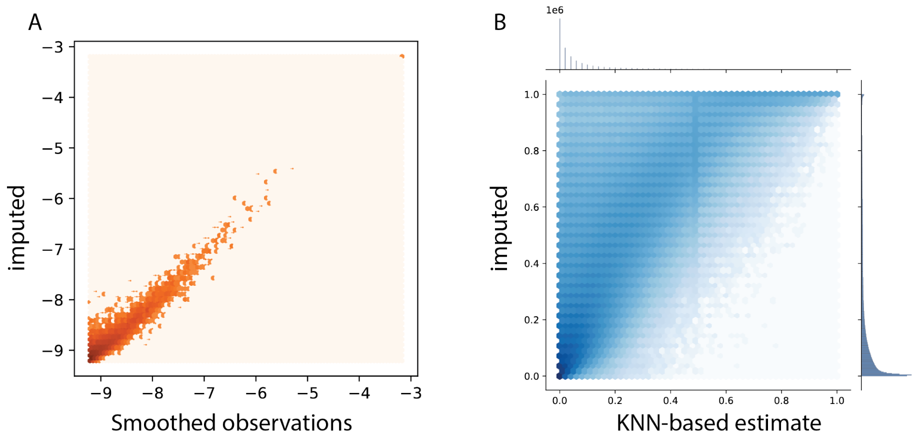

Figure S7: **Imputed values compared against smoothed observations.** Smooth averages of highly-similar cells (using 50 nearest neighbors in an independent low-dimensional space, computed separately for RNA and ATAC data) plotted against MultiVI-imputed values for expression (A) and accessibility (B).

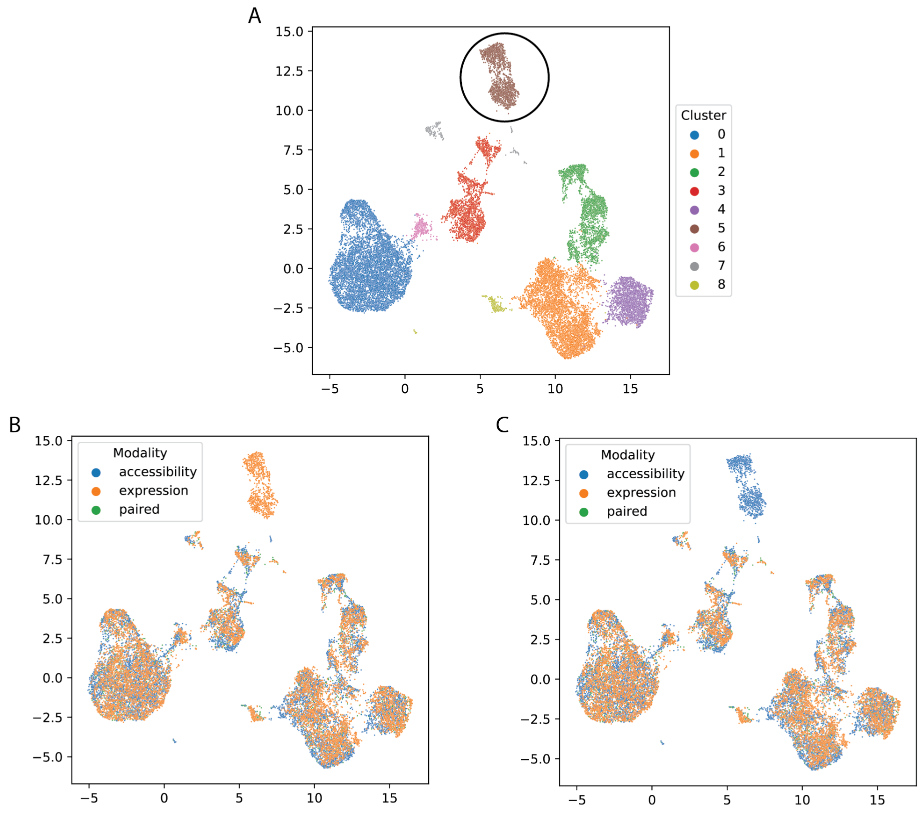

Figure S8: **UMAP visualizations of MultiVI's latent representation for 10x PBMC multiome dataset, with** 75% **of cells artificially corrupted.** A) Leiden clustering of the cells. B-C) modalities of the different cells after removal of all accessibility (B) or expression (C) data from the B-cell compartment (cluster 5).

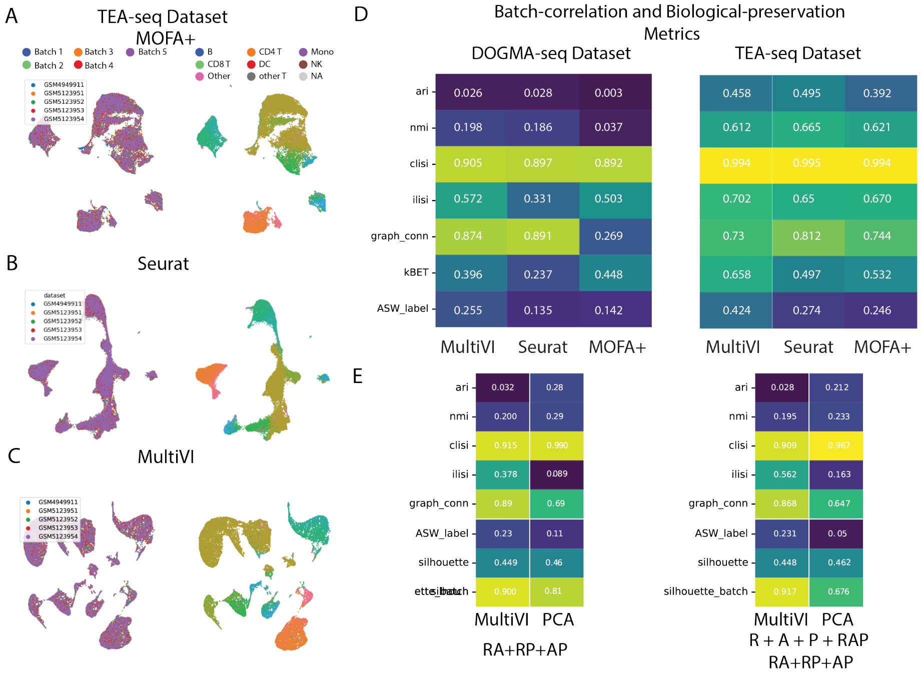

Figure S9: **MultiVI integrates transcriptional, chromatin accessibility and protein expression information into a meaningful latent space.** Latent representations for TEA-seq data set computed by MOFA (A), Seurat WNN (B) and MultiVI (C). In each panel, cells are color-coded by replicate (left), and cell type (right). D) Summary metrics describing batch correction and cell type identity preservation. E) Summary metrics describing modality integration for data sets containing different data modalities (portrayed in Figure 6E,F). R: transcriptional, A:chromatin, P:protein, RA:transc. and chrom., RP: transc. and prot., AP: chrom. and prot., and RAP: transc. and chrom. and prot.

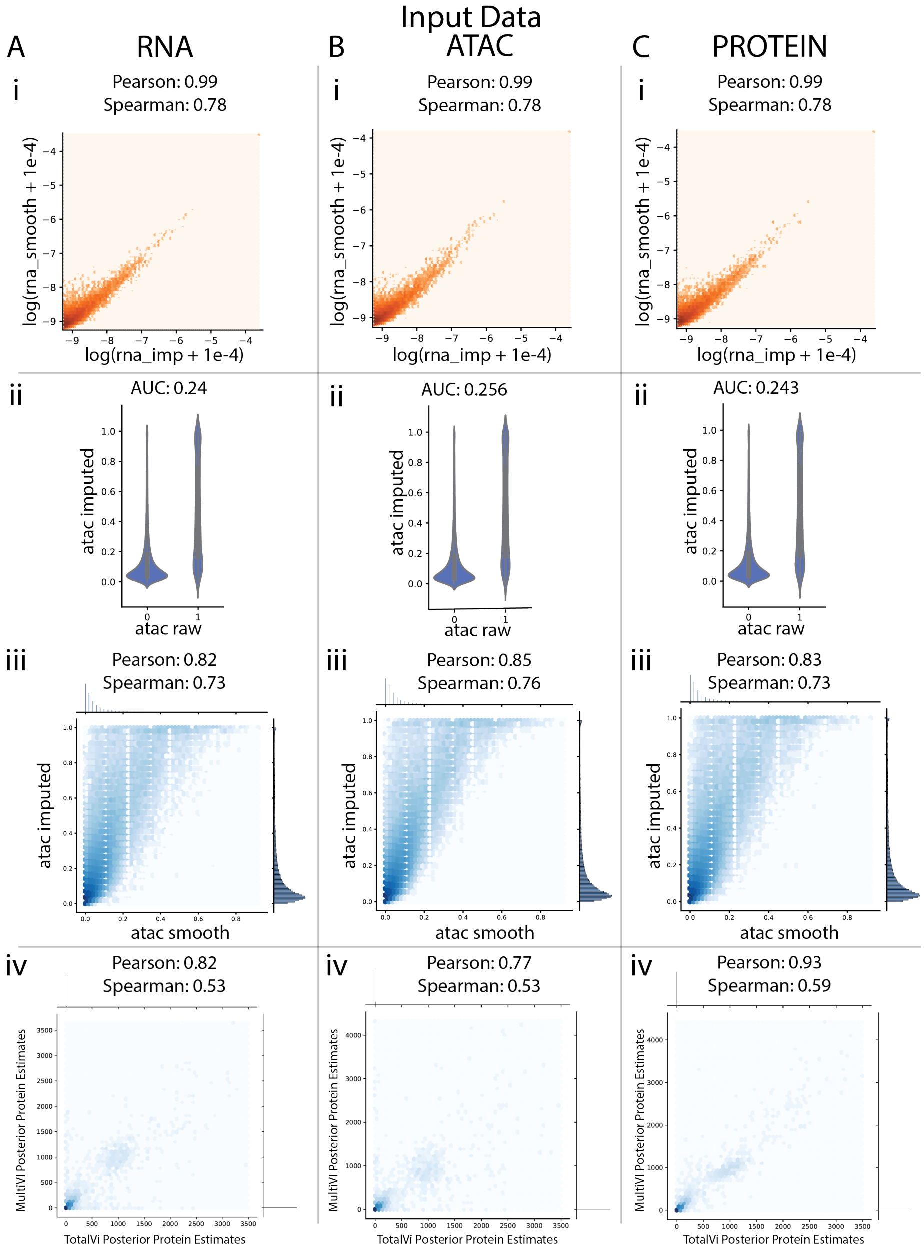

Figure S10: Caption in next page.

Figure S10: **MultiVI integrates transcriptional, chromatin accessibility and protein expression information, and imputes data missing in single modality data sets, generating uncertainty estimation**. MultiVI was trained using a DOGMA-seq data set in which information for RNA-seq, ATAC-seq, and CITE-seq is present for 8.3% of the cells, and only single-modality information is present for remainder (1/3 only RNA, 1/3 only ATAC, 1/3 only protein). Imputation of missing data in single modality cells is organized in three columns representing input data; i.e., RNA A), Chromatin Accessibility B), and Protein Expression C). For each of the corresponding input modalities, we impute the values of i) normalized RNA expression and compare it against smooth estimates of RNA expression (presented on a log scale ($\log(x+1e-4)$ for stability); ii) impute accessibility estimates and visualize them by their observed values; iii) impute accessibility estimates and visualize them against smooth accessibility estimates computed by averaging cells using Latent Semantic Indexing; iv) impute normalized foreground protein expression estimates and compared them against estimates computed using TOTALVI in which RNA and protein data is used as input to the model.
